# Prediction of a new ground state of superhard compound B_6_O at ambient conditions

**DOI:** 10.1038/srep31288

**Published:** 2016-08-08

**Authors:** Huafeng Dong, Artem R. Oganov, Qinggao Wang, Sheng-Nan Wang, Zhenhai Wang, Jin Zhang, M. Mahdi Davari Esfahani, Xiang-Feng Zhou, Fugen Wu, Qiang Zhu

**Affiliations:** 1 School of Physics and Optoelectronic Engineering, Guangdong University of Technology, Guangzhou 510006, China; 2 Department of Geosciences and Center for Materials by Design, Institute for Advanced Computational Science, State University of New York, Stony Brook, NY 11794, USA; 3 Skolkovo Institute of Science and Technology, Skolkovo Innovation Center, 3 Nobel St., Moscow 143026, Russia; 4 Moscow Institute of Physics and Technology, 9 Institutskiy Lane, Dolgoprudny city, Moscow Region 141700, Russian Federation; 5 Northwestern Polytechnical University, Xi’an 710072, China; 6 Department of Physics and Electrical Engineering, Anyang Normal University, Anyang, Henan Province 455000, China; 7 Peter Grünberg Research Center, Nanjing University of Posts and Telecommunications, Nanjing 210003, China; 8 School of Physics and Key Laboratory of Weak-Light Nonlinear Photonics, Nankai University, Tianjin 300071, China

## Abstract

Boron suboxide B_6_O, the hardest known oxide, has an *R*
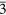
*m* crystal structure (α-B_6_O) that can be described as an oxygen-stuffed structure of α-boron, or, equivalently, as a cubic close packing of B_12_ icosahedra with two oxygen atoms occupying all octahedral voids in it. Here we show a new ground state of this compound at ambient conditions, *Cmcm*-B_6_O (β-B_6_O), which in all quantum-mechanical treatments that we tested comes out to be slightly but consistently more stable. Increasing pressure and temperature further stabilizes it with respect to the known α-B_6_O structure. β-B_6_O also has a slightly higher hardness and may be synthesized using different experimental protocols. We suggest that β-B_6_O is present in mixture with α-B_6_O, and its presence accounts for previously unexplained bands in the experimental Raman spectrum.

Superhard materials are used in many applications, from cutting, grinding and drilling tools to wear-resistant coatings^1^,^2^,^3^. However, most superhard materials^4^, such as diamond^5^ and cubic-BN^6^, are synthesized at high pressure, which makes them expensive, but some (boron allotropes, B_6_O, B_4_C) are thermodynamically stable already at ambient conditions. The hardness of α-B_6_O^7^ was reported to be in the range 30–45 GPa^8^,^9^, making it the hardest known oxide^9^,^10^,^11^.

Objects with icosahedral symmetry (*I*_h_) bear a special fascination because of incompatibility of fivefold symmetry with crystalline periodicity. The discovery of multiply-twinned particles B_6_O, an icosahedral packing of B_12_ icosahedra with *I*_h_ symmetry, had aroused interest^7^. Here we report the prediction of a new phase of B_6_O, with space group *Cmcm*, which we name β-B_6_O. This structure is energetically almost degenerate with α-B_6_O (and slightly more stable), is predicted to have a higher hardness, and actually corresponds to twinned α-B_6_O structure.

## Results

### Discovery of β-B_6_O at ambient conditions

**Figure 1:**
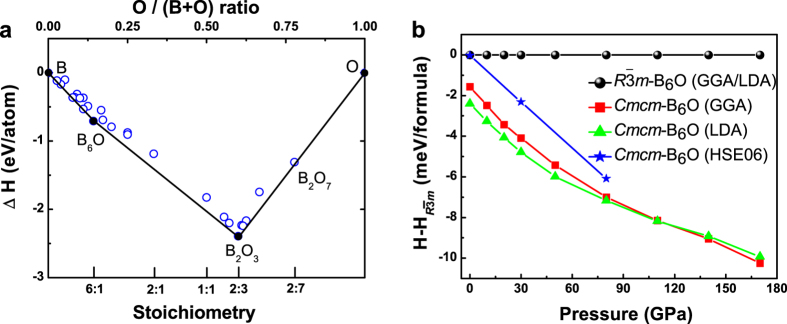
Stability of *Cmcm*-B_6_O. (**a**) Convex hull of the B-O system at ambient pressure. The solid (hollow) points represent the stable (metastable) structures. (**b**) Enthalpy difference between β-B_6_O and α-B_6_O, including zero-point energy.

Our variable-composition evolutionary searches expectedly find B_2_O_3_ and B_6_O to be the only stable compounds in the B-O system. Interestingly, there are also several compounds very close to stability - B_2_O_7_ (this is a 2D-form of B_2_O_3_ intercalated with oxygen molecules) and oxygen-deficient versions of B_6_O with B_6_O-like structures and compositions between B and B_6_O. To our surprise, *Cmcm*-B_6_O (β-B_6_O, see Table 1 for structural parameters), instead of the well-known *R*
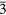
*m*-B_6_O (α-B_6_O)^7^,^12^,^13^,^14^, turned out to be the most stable structure at ambient pressure, as shown in Fig. 1; phonon calculations have confirmed its dynamical stability. Transmission electron microscopy recently confirmed its existence^15^. Structural parameters and some of the physical properties of β-B_6_O are shown in Table 1, in comparison with α-B_6_O and two related forms of pure boron.

In order to further compare the stability of β-B_6_O and α-B_6_O, we calculated their enthalpies as a function of pressure, as shown in Fig. 1b. We found that the enthalpy of β-B_6_O is lower than that of α-B_6_O at ambient pressure, but the energy difference is only about 1.8 meV/formula within the GGA (and almost degenerate within the HSE06 hybrid functional). As pressure increases, β-B_6_O becomes progressively more favorable than α-B_6_O, indicating that β-B_6_O might be more easily synthesized under pressure. The enthalpies of the two structures are so close that it makes us think: will the two structures coexist? what is their relationship? how to synthesize β-B_6_O? In order to answer these questions, we perform a detailed comparison of their structure, Raman spectra and phonon densities of states (PDOS).

### Comparison of crystal structures of α-B_6_O and β-B_6_O

**Figure 2:**
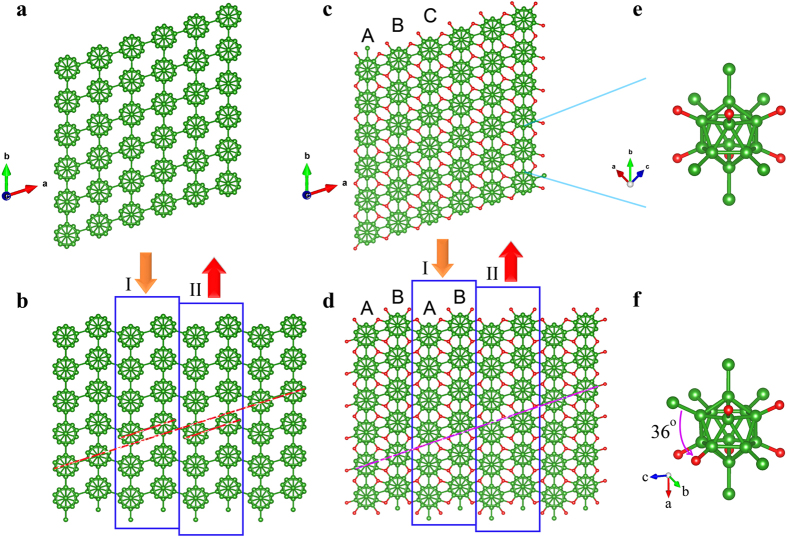
Crystal structures of (**a**) α-B, (**b**) *Cmcm*-B, (**c**) α-B_6_O, (**d**) *Cmcm*-B_6_O, and their local structures, (**e**,**f**) B_12_ icosahedra. Green (large) and red (small) spheres denote B and O atoms, respectively.

β-B_6_O structure has hexagonal close packing of B_12_ icosahedra (ABAB… stacking), while α-B_6_O is based on the cubic close packing (ABCABC… stacking) of B_12_ icosahedra, as shown in Fig. 2d,c. As is the case of hcp and fcc metals, twinning of α-B_6_O can produce local β-B_6_O stackings. It may also be possible to obtain β-B_6_O-like stacking faults by deformation of α-B_6_O, through plane slips. Most properties of these two phases are very similar: e.g. predicted volume per formula (*V*(α-B_6_O = 51.71 Å^3^/formula; *V*(*Cmcm*-B_6_O) = 51.69 Å^3^/formula), hardness (*Hv* (α-B_6_O) = 38 GPa; *Hv* (*Cmcm*-B_6_O) = 39 GPa), elastic moduli (Table 1), DFT band gaps (α-B_6_O has a 1.85 eV direct band gap, while *Cmcm*-B_6_O has a 1.81 eV indirect band gap).

Another interesting aspect is that if we remove the oxygen atoms from α-B_6_O and *Cmcm*-B_6_O, they turn into α-B^16^ and *Cmcm*-B, respectively (Fig. 2a,b). α-B and *Cmcm*-B are energetically nearly degenerate structures of boron at low pressure^17^, while *Cmcm*-B is a newly predicted structure^18^,^19^. As shown in Fig. 2b, displacements of layers I and II can transform this structure into α-B, and vice versa; *Cmcm*-B_6_O and α-B_6_O have a similar relationship (Fig. 2d). Furthermore, we found that the conversion of *Cmcm*-B_6_O and α-B_6_O will cause a deflection of B-O bond by 36°, as shown in their local structure (Fig. 2e,f).

However, it should be pointed out that it is not easy to change the stacking in covalent systems, though some examples are known – lonsdaleite (metastable “hexagonal diamond”) is formed in shocked cubic diamond. To obtain multiple polytypes, methods like physical vapor transport (PVT), also known as seeded sublimation growth, can be used: e.g., different polytypes of SiC were obtained using the PVT method^20^.

### Comparison of Raman spectra

**Figure 3:**
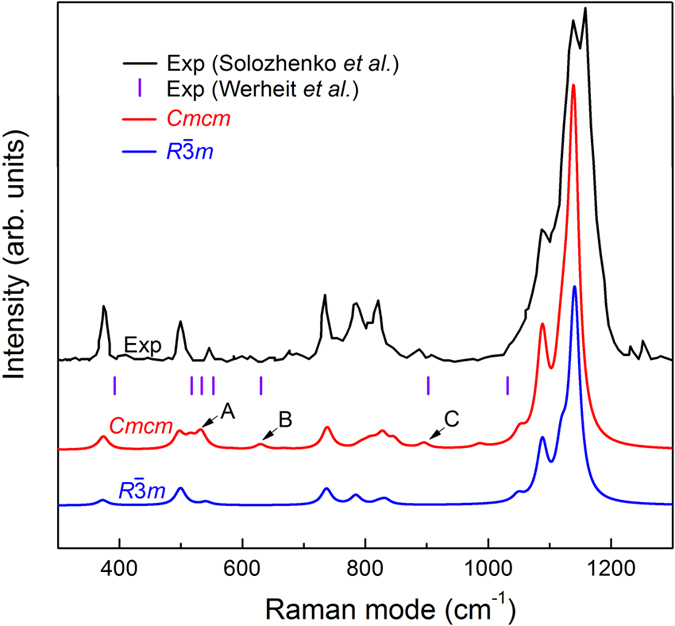
Raman spectra of B_6_O experimental spectrum of Solozhenko *et al.*^12^, Raman mode frequencies experimentally observed by Werheit and Kuhlmann^21^, and our calculated Raman spectra of *Cmcm*-B_6_O and α-B_6_O.

As mentioned above, β-B_6_O and α-B_6_O are energetically almost degenerate at zero pressure, structurally related and can coexist. To test the latter, we calculated their Raman spectra and compared with the experimental results^12^,^13^,^21^. In Fig. 3, the topmost curve is the Raman spectrum reported by Solozhenko *et al.*^12^; below it are the Raman frequencies reported by Werheit and Kuhlmann^21^ and marked by vertical bars (|). The two curves below are our Raman spectra of β-B_6_O and α-B_6_O. The experimental data correspond to normal isotopic abundance, and so do our calculations. Correspondingly, the atomic mass of boron in the calculations adopt the weighted average value, i.e. 10.811, which is based on the isotopic abundance of boron. As one could expect, the Raman spectra of β-B_6_O and α-B_6_O are similar and match perfectly with the experimental spectra. For example, the Raman modes at 374, 499, 541, 737, 785, 833, 1088, 1119, 1141 cm^−1^ are consistent with Solozhenko’s results^12^. However, there are four Raman modes which are unique for β-B_6_O. The first two are computed to be at 516 and 533 cm^−1^ (marked by letter A in Fig. 3), and Werheit and Kuhlmann indeed observed these two modes at 519 and 534 cm^−1^. The third mode is predicted to be at 630 cm^−1^ (marked with letter B), and Werheit and Kuhlmann have indeed observed a Raman-active phonon at 627 cm^−1^. We note that α-B_6_O does not have Raman-active modes between 570 and 700 cm^−1^, thus the one observed by Werheit and Kuhlmann at 627 cm^−1^ cannot come from α-B_6_O, but β-B_6_O. The fourth mode has the theoretical Raman frequency of 896 cm^−1^ (marked with letter C), while α-B_6_O has no Raman-active modes between 850 and 1000 cm^-1^. Thus, this mode is also unique to the β-B_6_O structure, and again seen in experiments: Solozhenko *et al.* observed Raman active phonon at 889 cm^−1^, and Werheit and Kuhlmann observed a Raman-active phonon at 902 (909) cm^−1^. Moreover, Wang *et al.* also observed similar Raman spectra in their B_6_O samples (see Fig. 1 in ref. 13). This analysis clearly shows that experimental samples contain β-B_6_O.

### Comparison of PDOSs and Gibbs free energy

**Figure 4:**
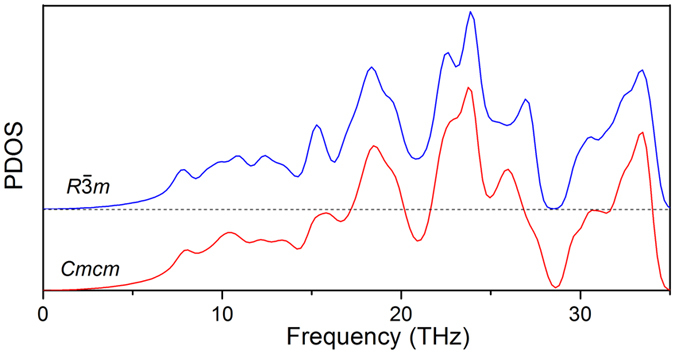
Phonon density of states of α-B_6_O phase and *Cmcm*-B_6_O phase at ambient pressure. For clarity, the PDOS of α-B_6_O was shifted.

Comparing phonon densities of states (PDOSs) of α-B_6_O and β-B_6_O at ambient pressure (Fig. 4), we once again see a great degree of similarity. In order to further confirm the stability of β-B_6_O, we have calculated the Gibbs free energy of β-B_6_O and α-B_6_O as a function of temperature, shown in Supplementary Fig. S2. We conclude that β-B_6_O remains more stable than α-B_6_O also when temperature is taken into account – and is even slightly stabilized by thermal effects.

Having found that β-B_6_O should be more stable than α-B_6_O, and demonstrated that the two phases actually coexist in experimental samples, we ask a question: why is the synthetic compound mostly α-B_6_O, instead of the more stable β-B_6_O? While there can be no definitive answer at this point, we suggest that this may be because of the use of α-rhombohedral-B (*R*
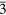
*m*)^9^ or β-rhombohedral-B (*R*
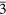
*m*)^22^ as a starting material (together with B_2_O_3_) for synthesis of rhombohedral-B_6_O. Hubert *et al.*^23^ used amorphous-B as the starting material, but they got not only B_6_O but also B_6_O twinning particles and some amorphous phases. And they observed a stacking of ABAB… around the planar defect, which is the same as β-B_6_O (We speculate it probably is β-B_6_O). To obtain β-orthorhombic-B_6_O, one would need to crystallize it *from the melt* (preferably at high pressure, to increase its thermodynamic advantage over α-B_6_O), or use other phases of boron as precursors. Using *Cmcm*-B would be ideal, but this phase (just 11 meV/atom higher in energy than α-B^19^) remains hypothetical, though likely to be eventually synthesized. Alternatively, the PVT method^20^ could be used.

In summary, to our big surprise, *ab initio* structure prediction calculations discovered a new ground state for the widely studied superhard compound B_6_O – our predicted β-B_6_O is more stable than experimentally known α-B_6_O. The two phases are polytypes and have nearly the same densities (β-B_6_O is slightly denser), energies (slightly lower for β-B_6_O), band gaps (slightly smaller and indirect, rather than direct, for β-B_6_O), hardnesses (β-B_6_O is slightly harder) and phonon densities of states, but have important differences in Raman spectra. By comparing calculated and experimental Raman spectra, we demonstrated that the experimental samples are actually a mixture of α-B_6_O and β-B_6_O. The discovery of β-B_6_O opens up new possibilities, in view of its greater stability, hardness and indirect band gap. Our findings also indicate possibilities of tuning the properties of B_6_O by obtaining phase-pure samples (probably not obtained to date), and the possibility of metastable oxygen-deficient compounds based on α-B_6_O or β-B_6_O - these can be obtained at high temperatures (where disordered oxygen vacancies will stabilize the structure) and low chemical potentials of oxygen.

## Methods

We used the *ab initio* evolutionary algorithm USPEX^24^,^25^,^26^,^27^ to search for thermodynamically stable B-O compounds and their structures at ambient pressure. This methodology has shown its predictive power in many studies (e.g., ref. 17 and 27, 28, 29). All structures were relaxed; structure relaxations and total energy calculations were done using density functional theory (DFT) within the generalized gradient approximation (GGA)^30^ as implemented in the VASP code^31^, with the projector-augmented wave method^32^. We used plane-wave kinetic energy cutoff of 600 eV, and sampled the Brillouin zone with uniform Γ-centered meshes of is 2π*0.07 Å^−1^ resolution within structure search, and 2π*0.04 Å^−1^ for subsequent highly precise relaxations and properties calculations. In order to confirm the relative stability of α-B_6_O and β-B_6_O, we used local density approximation (LDA)^33^ and HSE06 hybrid functional^34^. Phonon spectra was computed by PHONOPY^35^ and VASP, and Raman spectra were calculated using the Fonari-Stauffer method^36^. Hardness was calculated with Chen model^37^ and Lyakhov-Oganov model^38^. Elastic moduli were computed using Voigt-Reus-Hill averaging^39^.

## Additional Information

**How to cite this article**: Dong, H. *et al.* Prediction of a new ground state of superhard compound B_6_O at ambient conditions. *Sci. Rep.* **6**, 31288; doi: 10.1038/srep31288 (2016).

## Supplementary Material

Supplementary Information

## Figures and Tables

**Table 1:** Calculated structural parameters, hardness, elastic moduli and band gap of α-B, *Cmcm*-B, α-B_6_O, and *Cmcm*-B_6_O phases at ambient pressure.

Phase	α-B	*Cmcm*-B	α-B_6_O	*Cmcm*-B_6_O
Space group	*R* 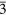 *m*	*Cmcm*	*R* 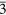 *m*	*Cmcm*
V, Å^3^/atom	7.248	7.262	7.387	7.384
Cell parameters	*a* = *b* = *c* = 5.050 Å, α = 58.04°	*a* = 4.883 Å, *b* = 8.852 Å, *c* = 8.064 Å α = β = γ = 90°	*a* = *b* = *c* = 5.153 Å, α = 63.10°	*a* = 5.393 Å, *b* = 8.777 Å, *c* = 8.736 Å α = β = γ = 90°
Atomic coordinates	B1(0.654, 0.010, 0.010) B2(0.630, 0.221, 0.221)	B1(0.000, 0.236, 0.568) B2(0.500, 0.937, 0.576) B3(0.823, 0.167,0.750) B4(0.797, 0.831,0.639) B5(0.682, 0.995,0.750)	B1(0.998, 0.998, 0.667) B2(0.676, 0.201, 0.201) O(0.622, 0.622, 0.622)	B1(0.000, 0.756, 0.588) B2(0.000, 0.549, 0.584) B3(0.165, 0.824, 0.750) B4(0.238, 0.155, 0.649) B5(0.334, 0.987, 0.750) O(0.000, 0.840, 0.439)
*Hv*_(Chen)_, GPa	39	35	38	39
*Hv*_(Lyakhov)_, GPa	33.0	32.7	31.6	31.7
*B*_0_, GPa	212(214^a^)	208	227(181^b^)	226
*G*, GPa	201	189	208	209
DFT band gap, eV	1.457	1.772	1.854^c^	1.805^c^

^a^Theory, reference 40.

^b^Experiment, reference 41.

^c^See Supplementary Fig. S1 for detailed band structures.
