# Supplementary material for: Prediction of a new ground state of superhard compound B6O at ambient conditions
Source: Sci Rep. 2016 Aug 8;6:31288. doi: 10.1038/srep31288 (PMC4976382; doi:10.1038/srep31288)
Supplement: Supplementary Information [file srep31288-s1.pdf]

Supplementary Information for

Prediction of a new ground state of superhard compound B_6O at ambient conditions

Huafeng Dong[1,4], Artem R. Oganov[2,3,4,5,*], Qinggao Wang[3,6], Sheng-Nan Wang[4], Zhenhai Wang[4,7], Jin Zhang[4], M. Mahdi Davari Esfahani[4], Xiang-Feng Zhou[4,8], Fugen Wu[1], and Qiang Zhu[4]

[1] School of Physics and Optoelectronic Engineering, Guangdong University of Technology, Guangzhou 510006, China

[2] Skolkovo Institute of Science and Technology, Skolkovo Innovation Center, 3 Nobel St., Moscow 143026, Russia.

[3] Moscow Institute of Physics and Technology, 9 Institutskiy Lane, Dolgoprudny city, Moscow Region 141700, Russian Federation

[4] Department of Geosciences and Center for Materials by Design, Institute for Advanced Computational Science, State University of New York, Stony Brook, NY 11794, USA

[5] Northwestern Polytechnical University, Xi'an 710072, China

[6] Department of Physics and Electrical Engineering, Anyang Normal University, Anyang, Henan Province 455000, China

[7] Peter Grünberg Research Center, Nanjing University of Posts and Telecommunications, Nanjing 210003, China

[8] School of Physics and Key Laboratory of Weak-Light Nonlinear Photonics, Nankai University, Tianjin 300071, China

*Corresponding author. E-mail: artem.oganov@stonybrook.edu (A.R.O.)

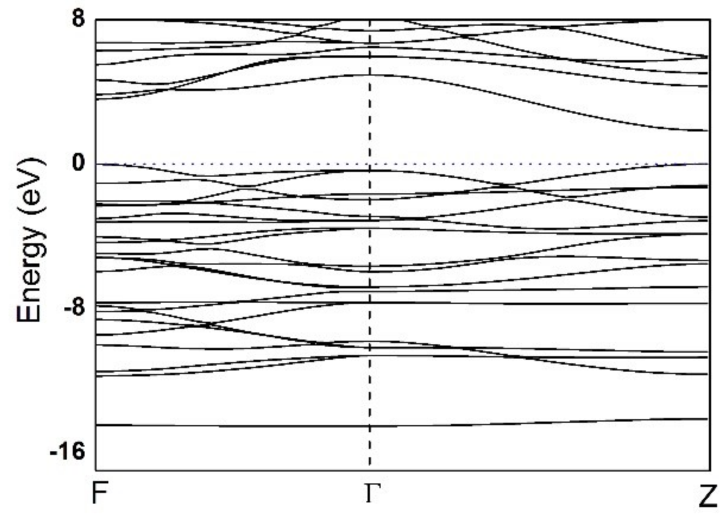

α-B_6O

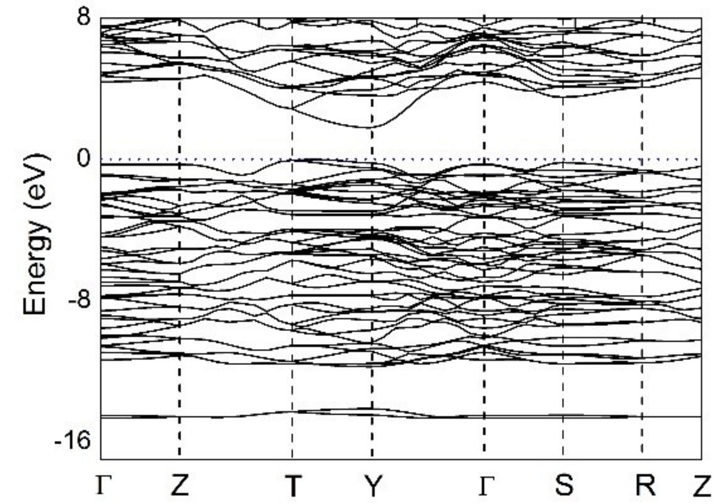

Cmcm-B_6O

Figure S1. Band structures of α-B_6O and *Cmcm*-B_6O.

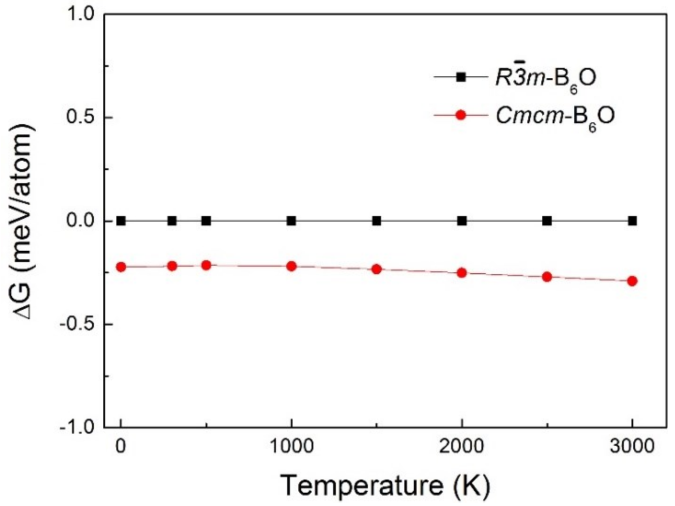

Figure S2. The Gibbs free energy (relative to α-B_6O) of *Cmcm*-B_6O as a function of temperature.
